# Complete Genome Sequence of the Radioresistant Bacterium Deinococcus aetherius ST0316, Isolated from Air Dust Collected in the Lower Stratosphere above Japan

**DOI:** 10.1128/mra.00836-22

**Published:** 2022-09-15

**Authors:** Katsuya Satoh, Kakeru Hagiwara, Kosuke Katsumata, Aya Kubo, Shin-ichi Yokobori, Akihiko Yamagishi, Yutaka Oono, Issay Narumi

**Affiliations:** a Department of Radiation-Applied Biology Research, Takasaki Advanced Radiation Research Institute, Quantum Beam Science Research Directorate, National Institutes for Quantum Science and Technology, Takasaki, Gunma, Japan; b Faculty of Life Sciences, Toyo University, Itakura, Gunma, Japan; c Graduate School of Life Sciences, Toyo University, Itakura, Gunma, Japan; d Department of Applied Life Sciences, Tokyo University of Pharmacy and Life Sciences, Hachioji, Tokyo, Japan; Wellesley College

## Abstract

Deinococcus aetherius ST0316 is a radioresistant bacterium that possess proficient DNA repair capacity. Here, we report the complete genome sequence of D. aetherius, which was obtained by hybrid assembly using short- and long-read sequencing. This sequence will be important information for elucidating the unique DNA repair mechanism of *Deinococcus* bacteria.

## ANNOUNCEMENT

The genus *Deinococcus* is well known as representing radioresistant bacteria, and about 100 species have been isolated from various environments (https://www.bacterio.net/genus/deinococcus). Deinococcus aetherius ST0316 was isolated as a UV- and gamma-radiation-resistant bacterium from air dust collected with membrane filters in the lower stratosphere above Japan (1, 2).

D. aetherius ST0316 cells were grown for 2 weeks at 30°C in membrane tryptone-glucose-extract (mTGE) broth (Difco) supplemented with 0.5% glycerol, 0.1 mM potassium dihydrogen phosphate, 5 μg/mL calcium (+)-pantothenate, and 14 μg/mL manganese(II) chloride tetrahydrate, harvested, and stored at −20°C. The genomic DNA was extracted with a Genomic-tip 100/G (Qiagen). For short-read sequencing, genomic DNA (25 ng) was sheared to approximately 500 bp using a Covaris S2 ultrasonicator. A library was prepared with a MGIEasy universal DNA library preparation set and a MGIEasy DNA adapters-96 (plate) kit (MGI). The quantity and quality of the library were validated using a Qubit 3.0 fluorometer, a Qubit double-stranded DNA (dsDNA) high-sensitivity (HS) kit (Thermo Fisher Scientific), a Fragment Analyzer system, and a dsDNA 915 reagent kit (Agilent). Circularized DNA and DNA nanoballs were constructed using a MGIEasy circularization kit and a DNBSEQ-G400RS high-throughput sequencing kit (MGI). DNBSEQ 2 × 200-bp paired-end sequencing was performed using a DNBSEQ-G400 sequencer (MGI). Adapter sequences were removed using Cutadapt v2.7 (3), and approximately 3.5 million read pairs (1.05 Gb) were collected from adapter-trimmed sequences using SeqKit v0.11.0 (4). Low-quality data (quality scores of <30 and read lengths of <127 bp) were removed using Sickle v1.33 (https://github.com/najoshi/sickle). For long-read sequencing, the same batched genomic DNA (1 μg) was fragmented and ligated to barcode adapters using a native barcoding expansion kit (EXP-NBD104; Oxford Nanopore Technologies). A library was constructed using a ligation sequencing kit (SQK LSK 109; Oxford Nanopore Technologies) and loaded on a GridION X5 R9.4.1 flow cell (Oxford Nanopore Technologies). Base calling was performed using Guppy v4.0.11+f1071ce (Oxford Nanopore Technologies). Adapter sequences and low-quality data (read lengths of <1,000 bp) were removed using Porechop v0.2.3 (https://github.com/rrwick/Porechop) and Filtlong v0.2.0 (https://github.com/rrwick/Filtlong), respectively. Then, DNBSEQ and GridION sequencing generated totals of 5,904,422 and 76,571 filtered reads, respectively. A hybrid *de novo* assembly was performed using Unicycler v0.4.7 (5) with default parameters. The assembled genomic data were validated using Bandage v0.8.1 (6) and CheckM v1.1.2 (7), respectively. Default parameters were used except where otherwise noted. The genome was 4,818,874 bp in total length, with an average GC content of 69.5% and mean coverage of 648-fold, and comprised a circular chromosome, three circular megaplasmids, and two circular plasmids (Table 1). The circularity was confirmed using Unicycler. The circular chromosome was rotated manually to the default starting gene *dnaA*. The genome was automatically annotated with the DFAST pipeline (8), as summarized in Table 1. The genome information will contribute to elucidating the unique DNA repair mechanism of *Deinococcus* bacteria.

**TABLE 1:** Genome features of Deinococcus aetherius ST0316

Parameter	Data for:					
	Chromosome	Megaplasmid pDAETH-1	Megaplasmid pDAETH-2	Megaplasmid pDAETH-3	Plasmid pDAETH-4	Plasmid pDAETH-5
Length (bp)	3,135,460	715,848	577,691	309,632	56,913	23,330
GC content (%)	69.8	68.9	69.7	68.6	69.1	65.1
No. of coding DNA sequences	3,237	676	539	334	81	26
No. of tRNAs	59	0	1	0	0	0
No. of rRNAs	9	0	3	0	0	0
DDBJ/ENA/GenBank accession no.	AP026560	AP026561	AP026562	AP026563	AP026564	AP026565

### Data availability.

The accession numbers for the complete genome sequence of D. aetherius ST0316 deposited in DDBJ/ENA/GenBank are listed in Table 1. The BioProject, BioSample, and DDBJ Sequence Read Archive (DRA)/NCBI SRA accession numbers are PRJDB14103, SAMD00518143, and DRA014620, respectively.
